# Bioreactor analyses of tissue ingrowth, ongrowth and remodelling around implants: An alternative to live animal testing

**DOI:** 10.3389/fbioe.2023.1054391

**Published:** 2023-02-20

**Authors:** Nupur Kohli, Konstantinos Theodoridis, Thomas A. G. Hall, Inigo Sanz-Pena, David C. A. Gaboriau, Richard J. van Arkel

**Affiliations:** ^1^ Biomechanics Group, Department of Mechanical Engineering, Imperial College London, London, United Kingdom; ^2^ FILM, National Heart & Lung Institute, Imperial College London, London, United Kingdom

**Keywords:** bone, mineralisation, osseointegration, preclinical, fixation, additive manufacturing, *ex vivo*, *in vitro*

## Abstract

**Introduction:** Preclinical assessment of bone remodelling onto, into or around novel implant technologies is underpinned by a large live animal testing burden. The aim of this study was to explore whether a lab-based bioreactor model could provide similar insight.

**Method:** Twelve *ex vivo* trabecular bone cylinders were extracted from porcine femora and were implanted with additively manufactured stochastic porous titanium implants. Half were cultured dynamically, in a bioreactor with continuous fluid flow and daily cyclic loading, and half in static well plates. Tissue ongrowth, ingrowth and remodelling around the implants were evaluated with imaging and mechanical testing.

**Results:** For both culture conditions, scanning electron microscopy (SEM) revealed bone ongrowth; widefield, backscatter SEM, micro computed tomography scanning, and histology revealed mineralisation inside the implant pores; and histology revealed woven bone formation and bone resorption around the implant. The imaging evidence of this tissue ongrowth, ingrowth and remodelling around the implant was greater for the dynamically cultured samples, and the mechanical testing revealed that the dynamically cultured samples had approximately three times greater push-through fixation strength (*p* < 0.05).

**Discussion:** *Ex vivo* bone models enable the analysis of tissue remodelling onto, into and around porous implants in the lab. While static culture conditions exhibited some characteristics of bony adaptation to implantation, simulating physiological conditions with a bioreactor led to an accelerated response.

## 1 Introduction

Secure implant fixation in bone is necessary for a successful outcome for a wide range of surgical procedures, including arthroplasty, spine fusion, fracture fixation, and dental implants, impacting millions of patients worldwide each year (Pabinger et al., 2015; Abbott et al., 2017; Pabinger et al., 2018; Borgström et al., 2020). The field is rife with innovation, including new implant technologies such as additively manufactured porous structures (Brogini et al., 2021; Munford et al., 2022) nano coatings (Bai et al., 2021) and biophysical stimulation (Soares dos Santos et al., 2016; de Sousa et al., 2021), as well as novel approaches to design (van Arkel et al., 2018; Wang et al., 2021) and innovation in surgical technique (Doyle et al., 2019; Doyle et al., 2020).

**FIGURE 1:**
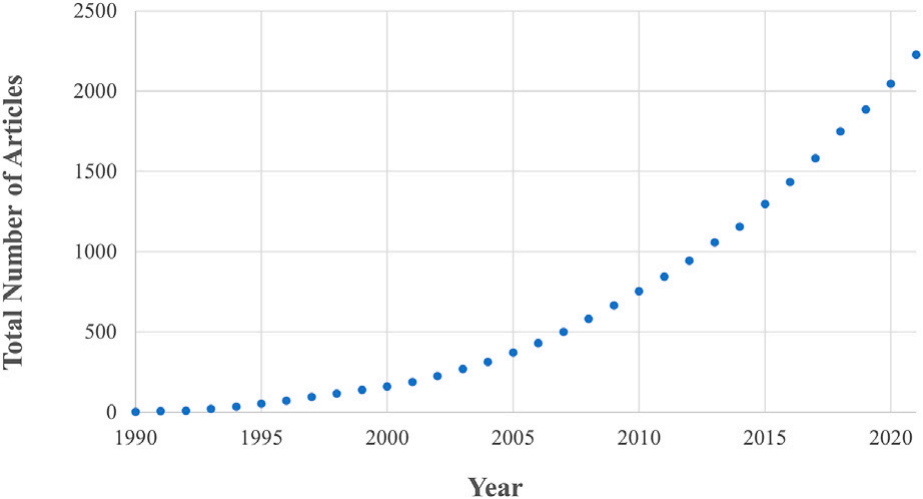
The number of *in vivo* animal studies published in the field of implant fixation as a function of publication year. Both the total number of articles and rate of publication is increasing. These data are the result of a Web of Science database search (17th August 2022) for: implant AND {fixation OR cementless OR osseointegration OR osteointegration OR [bone AND (“in-growth” OR “ingrowth” OR “in growth” OR “ongrowth” OR “on-growth” OR “on growth”)]} AND (porcine OR pig OR ovine OR sheep OR murine OR mouse OR mice OR rat OR bovine OR cow OR canine OR dog OR equine OR horse OR animal) AND (“*in vivo*” OR “*in-vivo*”) NOT review.

Fundamental to the success of new implant fixation technology is whether it drives favourable bone remodelling into, onto and around the implant. This remodelling process is driven by the interactions of three bone cell types, osteoblast, osteoclasts, and osteocytes, and is affected by the mechanical loading environment in addition to implant, patient, and surgical factors (Kohli et al., 2018). This complex interaction between different cell types, bone morphology and loading means that preclinical testing of new technology relies of on live animal testing. *In vivo* animal research has rapidly gained traction since the 1990s and is now considered a gold standard in the field, leading to an ever-increasing animal burden. Currently, ∼200 articles are published every year in the field of implant fixation alone (Figure 1) with >60 animals experimented on per paper (Öztürk and Ersan, 2020).

*Ex vivo* models of implant fixation could be a route to reduce this burden: many consider this to be replacement as the 3Rs principle (Replacement-Reduction-Refinement) is associated with live animal research. But even if not considered replacement, the approach still contributes greatly to reduction and refinement by enabling multiple samples to be obtained for each animal sacrificed, reuse of material that might otherwise be wasted, and by refining the protocol to eliminate all experimentation on the animal while it is living. There is even scope to use human tissue by utilising waste from routine surgical procedures, such as femoral heads resected during hip arthroplasty (Swarup et al., 2018; Styczynska-Soczka et al., 2021).

Early research has demonstrated that *ex vivo* bone can grow and adapt to mechanical loading in a bioreactor setup (Jones et al., 2003; Davies et al., 2006; David et al., 2008; Vivanco et al., 2013; Birmingham et al., 2015; Birmingham et al., 2016). More recent research has demonstrated material and cell transfer to an implant surface (Dua et al., 2021; Zankovic et al., 2021) and that is possible to maintain viability in samples as large as human femoral heads (Swarup et al., 2018; Styczynska-Soczka et al., 2021), but it is still not clear if the *ex vivo* bone can be used to explore the early stages of implant bone ingrowth into porous implants. Nor is it clear if the full remodelling pathways of bone are active. For example, osteoclasts survive for ∼2 weeks (Owen and Reilly, 2018), and so if new cells do not differentiate, one would expect a bias for growth driven by osteoblast activity in experiments lasting 3 weeks or more. Given that bone growth is considered the favourable outcome for new technologies, a meaningful preclinical test must also allow for the possibility of bone resorption.

The aim of this research is to analyse whether *ex vivo* bone models can be used to assess bone remodelling into, onto and around new implant fixation technology. Porous stochastic titanium scaffolds were used as an example implant technology given the large research interest in these structures for arthroplasty implants (Ghouse et al., 2019; Reznikov et al., 2019; Dion et al., 2020; Brogini et al., 2021; Munford et al., 2022), and two culture conditions were explored to understand the effects of research design on *ex vivo* bone remodelling and implant bony ingrowth.

## 2 Materials and methods

### 2.1 Bone sample preparation

**FIGURE 2:**
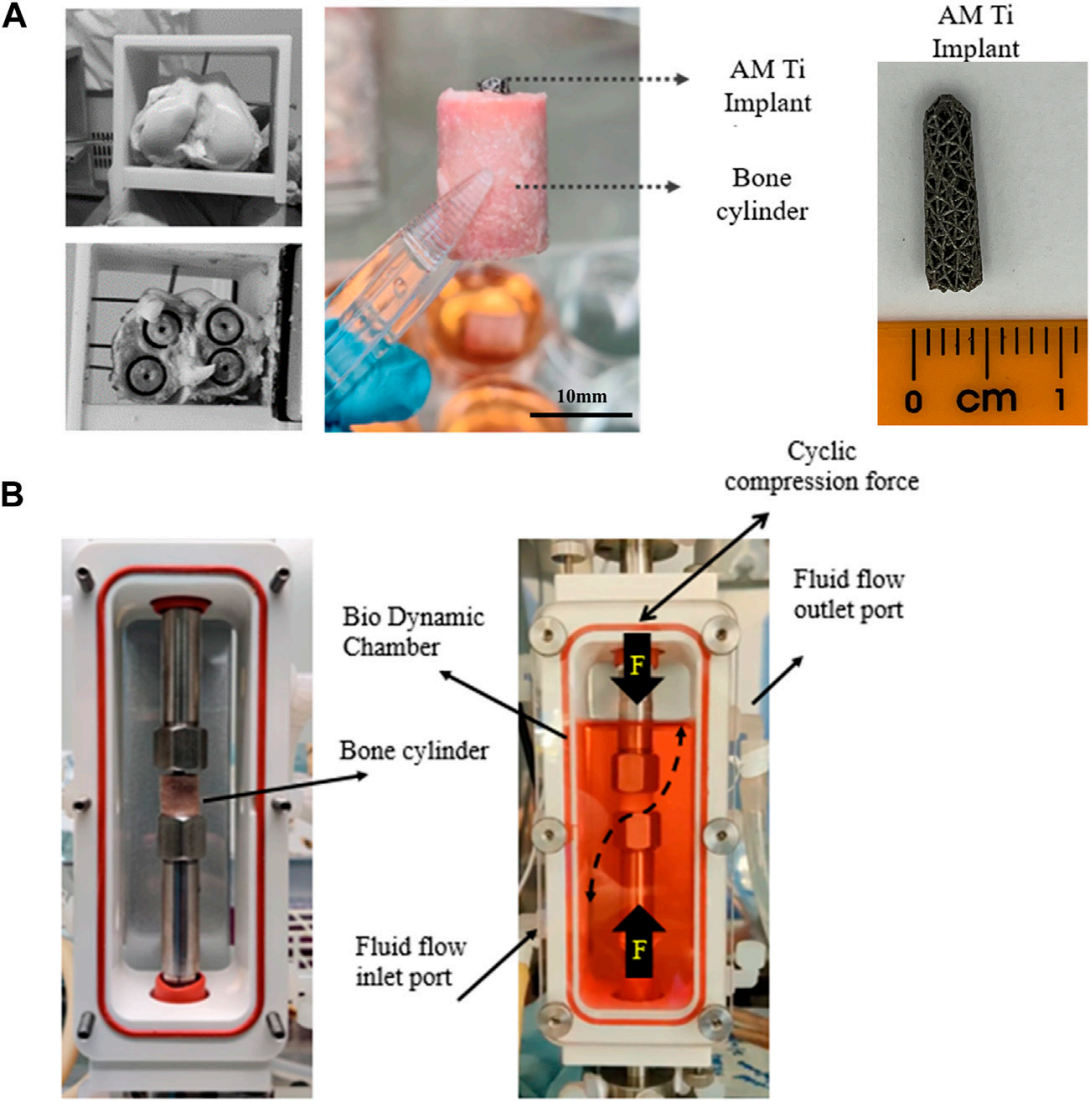
**(A)** Extraction of 4 trabecular bone cores from porcine femoral condyles and preparation for implantation with a tapered porous titanium peg. **(B)** For dynamic culture, the implanted bone cores were fixed within the biodynamic chamber of the bioreactor.

The research was registered annually in accordance with the host institution’s policy for use of animals in research, with all tissue sourced as a waste product from pigs that were sacrificed for unrelated reasons. Twelve femoral trabecular bone cores were obtained from healthy large white pigs (∼70 kg, Royal Veterinary College, UK), within 4h *post-mortem*. Briefly, hind limbs were transported on ice to the lab immediately following sacrifice. The limbs were transected at the mid-femur and mid-tibia and dissected free of soft tissue while preserving the knee capsule. The knee was moved into a sterile hood and placed in a container filled with 70% ethanol for 15 min, followed by a wash in Dulbecco’s Phosphate-Buffered Saline (1x DPBS, Thermo Fisher Scientific, United States) containing 5% antimicrobial solution (Penicillin-Streptomycin-Amphotericin B, MP Biomedicals, Gibco™, Thermo Fisher Scientific, United States) for 5 min. The knee capsule was dissected, and the distal femur resected with a sagittal saw to expose the trabecular bone, guided by an autoclaved cutting guide, akin to the distal femoral resection in total knee arthroplasty. A second guided cut was made 16 mm proximal to the first. Bone cylinders (Ø12 mm, height 16 mm, Figure 2A) were then extracted with a diamond hole saw followed by a Ø2.5 mm centred drill to insert the implants and placed in 50 mL tubes containing PBS with 1% antimicrobial solution. All procedures were done at low speed/rpm with constant irrigation of cold sterile phosphate buffered saline (PBS) to reduce heat generation during cutting.

### 2.2 Implant design and insertion

Tapered stochastic porous titanium implants of base Ø3 mm, taper angle 1.7° and length 13 mm (Figure 2A) were additively manufactured from commercially pure titanium powder (ASTM B348 Grade 2 spherical powder, Ø15-45 μm, Carpenter Additive, United States) using a powder bed fusion system (AM250, Renishaw, UK). The layer thickness was set to 50 μm and laser parameters were controlled in accordance with previous research (Ghouse et al., 2017; van Arkel et al., 2018), which characterized the properties of the stochastic porous structure used: apparent modulus 600 MPa, yield strength 8 MPa and 90% porosity (van Arkel et al., 2018). The implants were removed from the substrate, rinsed, cleaned ultrasonically, autoclave sterilised, and then press fitted into to the centre-drilled holes of the bone cylinders, leaving ∼2 mm of the top of the implant exposed. The implanted cylinders were transferred into 6-well tissue culture plates containing 10 mL of pre-warmed (37°C) culture medium, consisting of Dulbecco’s Modified Eagle’s Medium (DMEM/F-12, HEPES, Gibco™, Thermo Fisher Scientific, United States), 10% Fetal Bovine Serum (FBS, Gibco™, Thermo Fisher Scientific, United States) and 1% antimicrobial solution. The bone cylinders were incubated overnight (37°C, 21% O_2,_ and 5% CO_2_) prior to bioreactor setup.

### 2.3 Culture conditions

The implanted bone cylinders were divided into static (n = 6) and dynamic (n = 6) groups; the former used as controls.

#### 2.3.1 Static culture

Static samples were placed in a 6-well plate with fresh culture medium and cultured for 21 days. The media were changed at least every 3 days. To assess mineralising bone surfaces, fluorochrome labels were added to the media on day 7 (Calcein, Sigma-Aldrich) and 14 (Alizarin Red, Sigma-Aldrich), both at a concentration of 50 μg/mL and replaced with fresh media after 24 h.

#### 2.3.2 Dynamic culture

Dynamic samples were placed within a perfusion bioreactor (Figure 2B, Electroforce Biodynamic 5100, TA Instruments, Waters, UK). Chambers were filled with 150 mL of culture medium and connected to a reservoir filled with a further 100 mL of culture medium *via* platinum-cured silicone tubing and a peristaltic pump (Masterflex, Cole-Parmer, UK). Fluid flow was induced by circulating the media at a continuous rate of 8 mL/min.

Cyclic compressive loading was applied to the exposed top of the implant to model *in vivo* loading of press-fit pegs during daily activity, e.g., for femoral (Berahmani et al., 2015) or glenoid (Geraldes et al., 2017) arthroplasty components. The resulting axial displacement of the tapered peg into the straight sided drilled hole generates both tensile hoop stress and compressive radial stress in the bone. The loading was applied by the actuator *via* the biodynamic compression platens: implants were preloaded to 5 N, and then 30 μm cyclic compressive displacement (sinusoidal wave) at 1 Hz was applied for 300 cycles/day. The magnitude of the displacement was based on the level of interfacial micromotion consistently associated with osseointegration *in vivo* (Kohli et al., 2021). The resulting force was measured with the machine’s load cell and recorded. Fluorochrome labels were added on day 7 and 14 as per the static culture.

### 2.4 Imaging and mechanical analyses

Post culture, static, and dynamic bone cores were washed with PBS, sliced with a thickness of ∼4 mm below the top surface of the bone using a low-speed cutting saw (IsoMet 1000, Buehler, Lake Bluff, IL, US), and fixed in 10% neutral buffered formalin for 24 h. Once fixed, the samples were washed again with PBS and kept in the dark for further analysis. The bulk of the samples (cylinders of ∼12 mm height) were used for different imaging analyses and the tops for destructive mechanical testing.

For the imaging analyses, n = 3 dynamic and n = 3 static samples were imaged as cylinders, and 3 of each were resin embedded and sectioned. The latter was achieved by dehydrating samples in graded methylated spirits (50%, 75%, 85%, 95%, and 2% × 100%). Dehydrated samples were immersed in chloroform for 24 h, and then chloroform was switched to methyl methacrylate resin (LR White) for a further 72 h. After resin saturation, an accelerator was added to polymerize and block the specimens at −20°C. The polymethyl methacrylate blocks were sectioned parallel to the axis of the cylindrical implant, using a water-cooled bandsaw (Model 311, Exakt, Ger.). Sections were grinded (Model 400 CS, Exakt, Ger.) and polished to a final thickness of 50 μm/slice. Some of the slices remained unstained for fluorescent imaging, and the rest were further processed for histology.

#### 2.4.1 Widefield fluorescence microscopy

**FIGURE 3:**
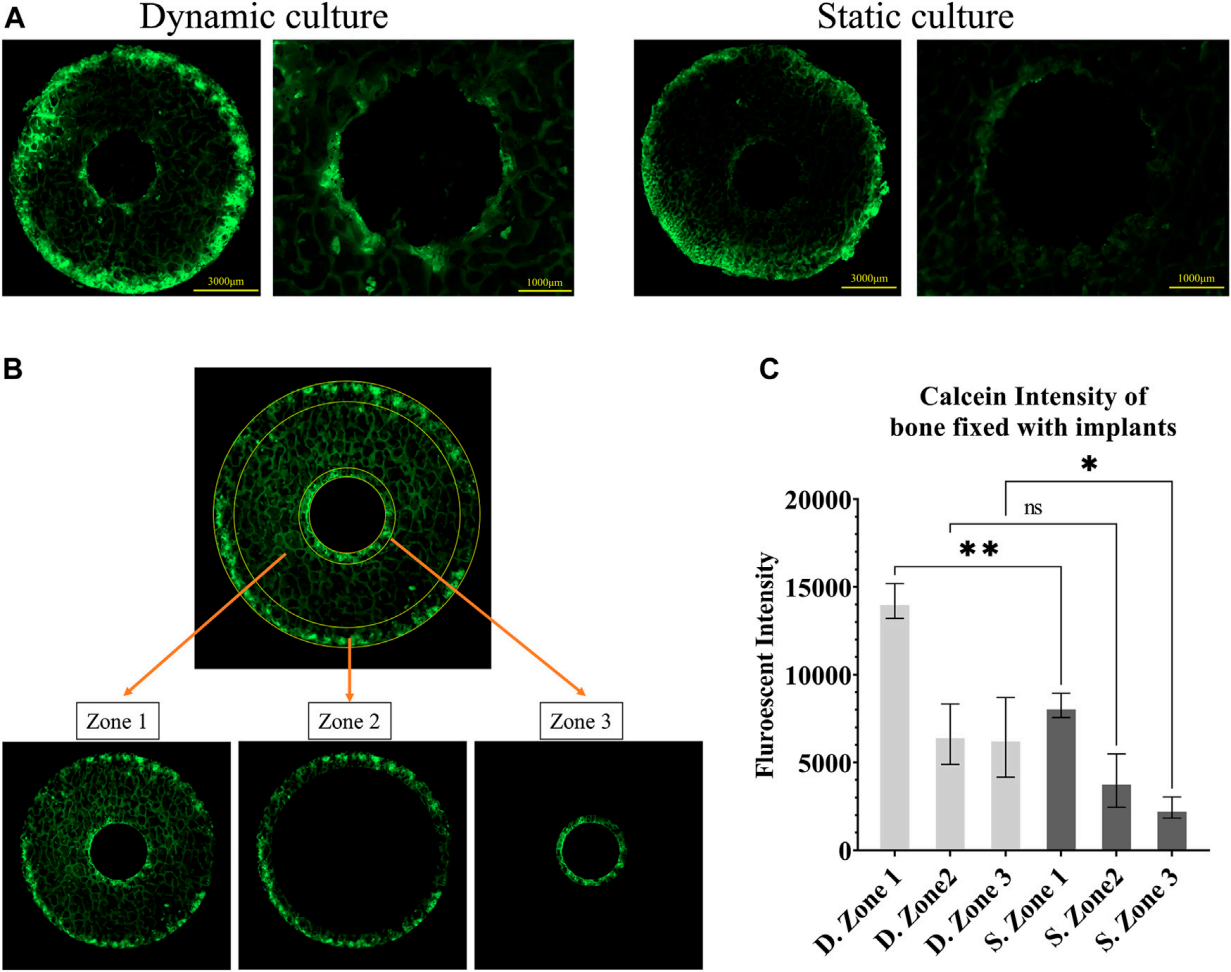
Fluorescent images of calcein administered on the seventh day **(A)** Representative images from static and dynamic cultures showing the top surfaces of the bone cylinders, assessed with widefield microscopy at λex495/λem515. **(B)** The three regions of interest that were quantitively analysed: zone 1, the whole bone cylinder, zone 2, the outer bone perimeter, and zone 3, the bone adjacent to the implant. **(C)** Analysis of calcein fluorescent intensity, showing mineralisation accumulation, comparing these 3 zones in static and dynamic culture samples. Values are means of n = 3 (±SD); *p <0.05, **p <0.01, ***p <0.001.

The top surface of the bone cylinder was scanned with a ×5 objective (Axio Observer, ZEISS, Oberkochen, Ger.) using a tile and stitching method (ZEN Pro, ZEISS, Oberkochen, Ger.) (Figure 3A). Wavelengths of λ_ex_495/λ_em_515 and λ_ex_545/λ_em_580 were set for calcein and alizarin red, respectively.

Bone remodelling activity around the implant was first quantified by measuring calcein fluorescent intensity on the top surface of the cylindrical samples as a measure of total mineral accumulation. We used Fiji/ImageJ (National Institutes of Health (NIH), United States) to calculate the mean intensity in three zones: the full cross-section (zone 1), the outer ring of the bone cores that were damaged during cutting (zone 2) and most exposed to the fluid flow, and an inner ring immediately adjacent to the implant (zone 3) (Figure 3B). Bone labelling found within the porous implant was excluded from this analysis of bone adaptation by designing a circular mask centred on the implant hole and removing all stained areas within that circle.

**FIGURE 4:**
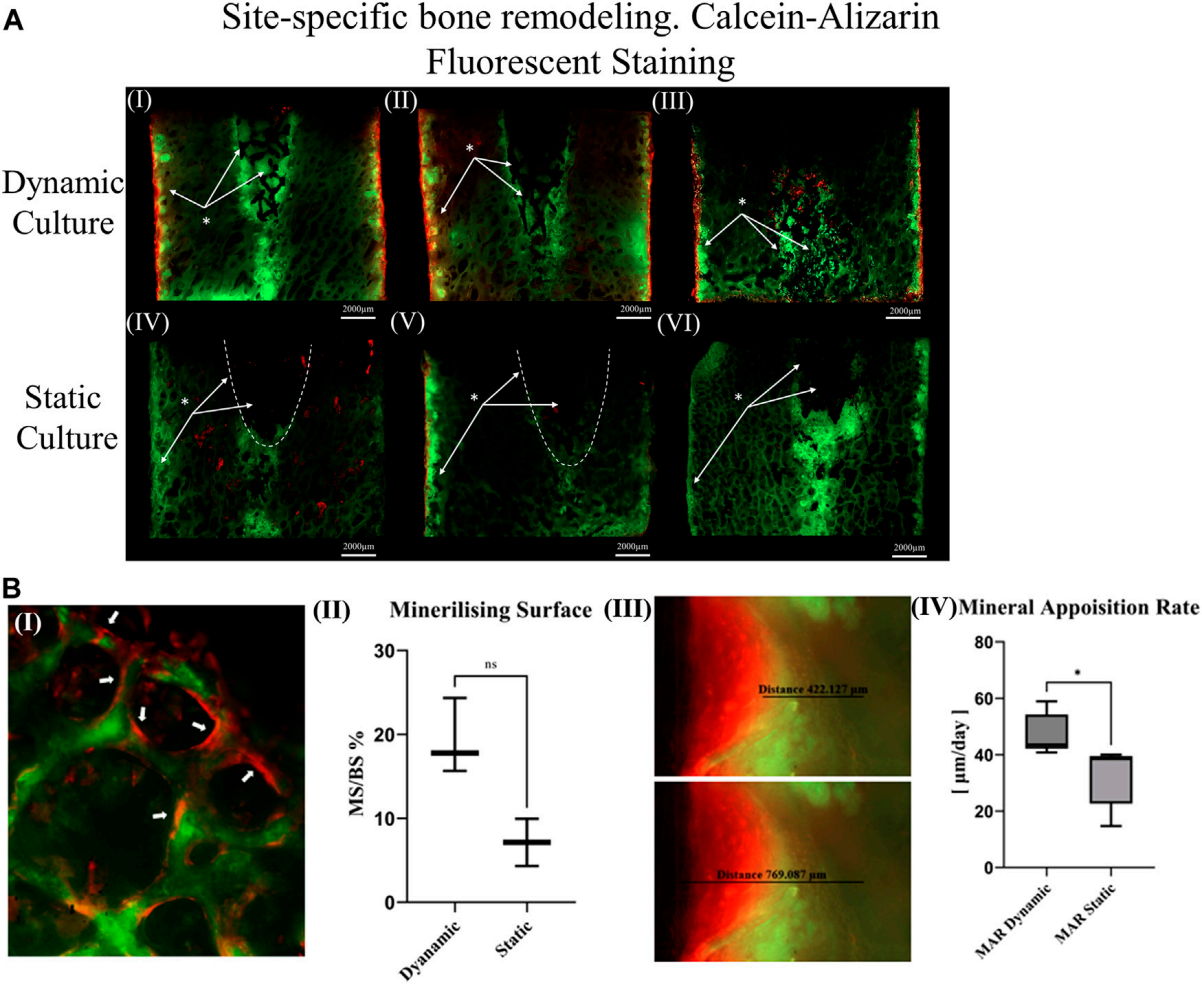
**(A)** Thinned sections (50 μm/slice) of the bone cylinders stained with calcein on the seventh day and Alizarin red on the 14th day for three dynamic culture replicates (I-III) and three static culture replicates (IV-VI) **(B)** Representative images from dynamic samples. (B.I) Mineralizing surfaces (MS) evaluated from the bone struts found on the outer perimeter of the bone cylinders on the top surfaces. (B.II) Measurements of the MS/BS expressed in percentage. (B.III) Mineral apposition rate found on the sides of the bone cylinders (thinned sections) and (B. IV) mean distance of both labels divided by seven expressed in μm/day. Values are means of n = 3 (±SD); *p <0.05.

The top surfaces of the cylindrical samples were also used to measure the mineralizing surface (MS) as a percentage of the bone surface (BS) in regions where both fluorochrome stains were detected. Stained surfaces were annotated in QuPath (Bankhead et al., 2017), and the day 7 surface was divided from the day 14 surface (Figure 4BI).

Unstained resin-embedded sections were also analysed with fluorescent microscopy. From each section, bone labelling was quantified. The mineral apposition rate (μm/day) was evaluated on the sides of the bone cylinder by finding the mean distance between both labels (Figure 4B. III) divided by 7 (the time interval between the administration).

#### 2.4.2 Scanning electron microscopy

Morphology distribution of new bone tissue formation was observed by visualizing the surface of explanted porous implants with Scanning Electron Microscopy (SEM). Samples were fixed with 4% v/v glutaraldehyde, rinsed with PBS, and then dehydrated in increasing concentrations of ethanol series (30%–100% v/v). The samples were dried, sputter coated with a 15 nm layer of chromium, and observed under SEM-EDS (Mira, TESCAN, Cz.) at an accelerating voltage of 15 kV. Resin embedded sections were also visualized under SEM to assess tissue formation and distribution within the porous architecture of the implant using the Back Scatter Electron (BSE) technique.

#### 2.4.3 Micro-computed tomography

Samples were dehydrated in series of ethanol (25%, 50%, 75%), for 2.5 h per ethanol concentration and immersed in Phosphotungstic acid hydrate solution for 24 h to facilitate high contrast X-ray visualization. This solution comprised of 1% (w/v) Phosphotungstic acid (PTA, Sigma-Aldrich, UK), water solution (30 mL), all mixed with 70 mL absolute ethanol, to make 0.3% PTA in 70% ethanol (Metscher, 2009). Micro-CT scans (Xradia 510 Versa, ZEISS, Ger.) were acquired at a Zeiss at a voltage of 140 kV and 70 μA, without a filter and full rotation of 360°. The pixel size was set to 4.11 μm. All micro-CT scans were re-assembled as Z-stacks in Icy (De Chaumont et al., 2012). All Z-stacks were then converted for segmentation using Imaris software (Bitplane, Zurich, Switz.). The volume of the segmented tissue and titanium construct were calculated as to the truncated cone. Newly formed tissue was expressed as a percentage of the pore void, and 3D rendered images of the scanned specimens were created to represent the total tissue distribution.

#### 2.4.4 Histology

Resin-embedded sections were stained with toluidine blue and paragon stain for histological analyses. The cross-sectional slides of the bone tissue and the implant were digitised at ×5 objective and stitched using the tile method of the ZEN Pro software.

#### 2.4.5 Mechanical analysis

The characteristic loading times, $T_{load}$ , for the cyclic loading applied in the dynamic culture were calculated in accordance with the method established by Scheiner et al. (2016):

$$T_{load}=\frac{\left|Q\right|}{\left|\dot{Q}\right|}$$

where Q is the force measured by the load cell.

Post testing, the mechanical strength of the bone-implant interface was measured with push-through testing in a material-testing machine (Model 5565, Instron, UK). The top sections of the bone cylinders (height ∼4 mm) were placed on a compression platen with a central Ø8 mm hole (Figure 8A). A top compression platen of less than Ø3 mm (smaller than the implant) was brought into contact with the exposed implant (∼2 mm), before compressing the implant at a displacement rate of 1 mm/s. Force–displacement data were recorded, and the fixation strength was calculated by dividing the load at the first peak by the implant-bone contact area (a truncated cone).

### 2.5 Statistical analysis

GraphPad Prism software (version 9.4.0; GraphPad Software, San Diego, CA, United States) was used for the statistical analysis. Multiple comparisons between groups were performed using one-way ANOVA, followed by *post hoc* Tukey’s test. Differences between means were considered statistically significant when *p*) < 0.05 (∗), *p* < 0.01 (∗∗), *p* < 0.001 (∗∗∗). Data were presented as mean ± SD for N = 3 biological replicates for imaging analyses and N ≥ 5 for mechanical testing.

## 3 Results

### 3.1 Bone formation labelling—widefield analysis

Calcium mineralisation was detected when assessing the top surfaces of the cylindrical samples. Day 7 fluorochrome intensity was affected by the culture conditions (Figures 3A, B). Mean intensity across the full cross section was 172% greater for the dynamic samples compared to the static controls (*p* = 0.003, Figure 3C). When examining only the bone adjacent to the implant, the intensity was found to be 264% greater for the dynamic samples (*p* = 0.045, Figure 3C). Additionally, mineralised surfaces, stained with Alizarin red after 14 days of culture, were observed on the outer edges of the bone cylinders. Increased mineralised surface at the circumference of top surfaces for the dynamic samples was observed but did not reach statistical significance (*p* = 0.055, Figure 4B. I, II).

Sectioned images revealed calcium deposits within the porous implants for dynamically cultured samples, with less stain found within statically cultured implants (Figure 4A). Some calcium deposits were also observed in the intact bone below the implant’s push-in direction for both culture conditions. Mineralised surfaces stained with Alizarin red after 14 days of culture, were only observed to the outer edges of the bone cylinders and not inside the porous implants, consistent with the observations made from the cylindrical images. Analysis of these mineralising surfaces in the sectioned surfaces revelated that mineral apposition rate was 45% greater for the dynamic samples (*p* < 0.05).

### 3.2 Macroscopic characteristics and bone ongrowth—SEM analysis

**FIGURE 5:**
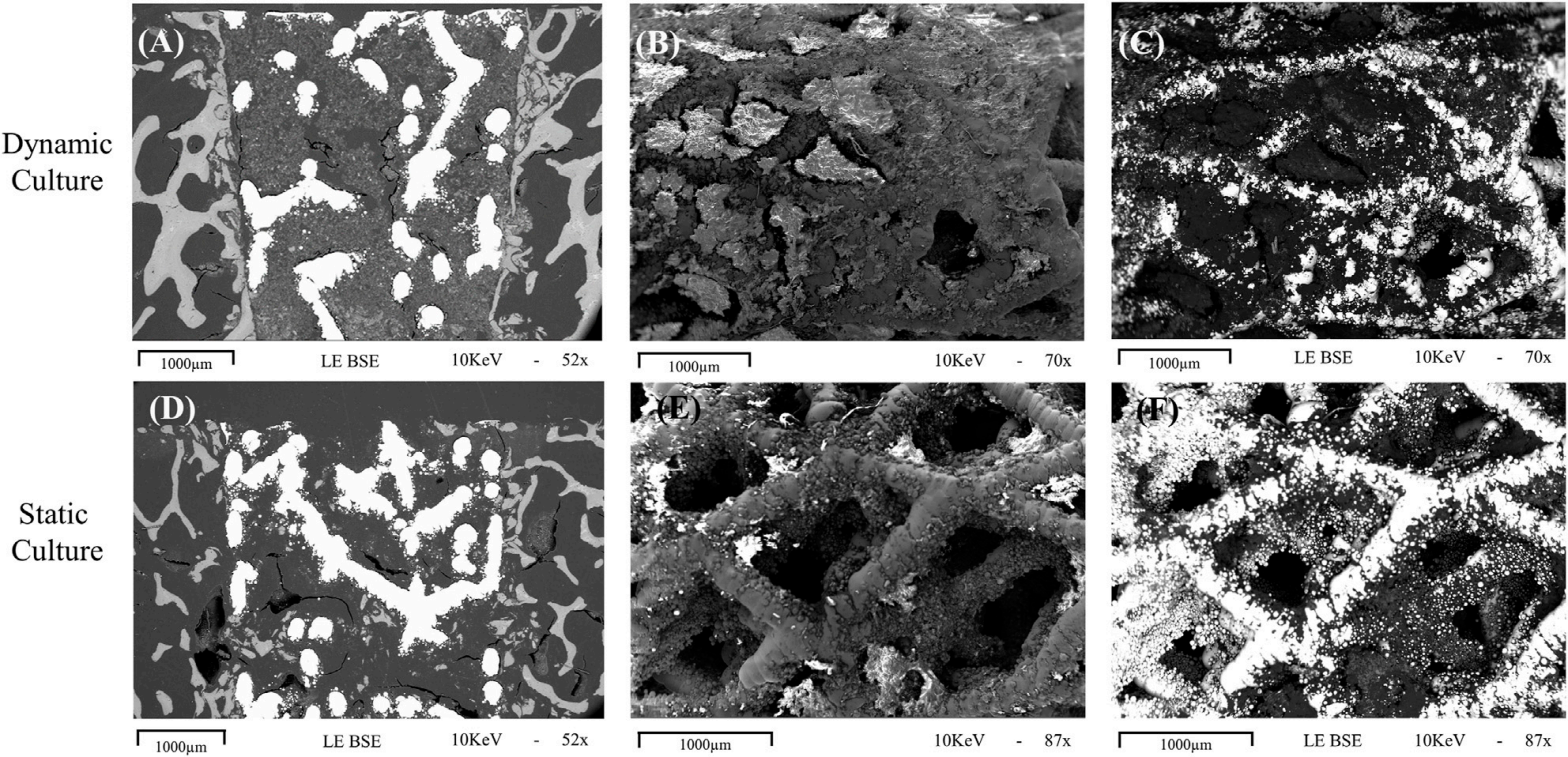
Representative images obtained with scanning electron microscopy for dynamic (top row, **(A-C)** and static (bottom row, **D-F**) cultures **(A, D)** Cross sections of the implant *in situ*, taken with the BSE technique showing evidence of mineralisation within the implant pores, with more seen in the sample taken from dynamic culture. **(B, C)** macroscopic images taken with standard and BSE methods, respectively, showing the outer cylindrical surface of an explanted implant with abundant tissue formation on the surface and within the pores. **(E, F)** standard and BSE images of the outer surface of an explanted statically cultured implant also showing tissue formation on the surface, albeit less than that seen for the dynamic culture. 1000 μm scalebars are shown.

Images from the thinned cross-sectional slides of dynamic samples revealed the most backscatter signal within the implant’s pore network (Figure 5A), indicative of the newly formed mineralized tissue. Some back-scatter signal was also detected within the pore network of the implant or at the bone-implant interface in static samples (Figure 5D).

For the explanted cylindrical implants, the surfaces and the first layers of the porous structure inside pores were visible in the SEM (Figure 5). For the dynamic samples large amounts of extracellular matrix was detected on the struts of the scaffolds, and inside the pores, bridging them with a newly formed tissue network (Figures 5B, C). The static culture also led to tissue formation in some pores, but not yet enough to develop abundant tissue formation on or within the pore network (Figures 5E, F). EDS analysis revealed the presence of both calcium and phosphorus in this on-grown tissue.

### 3.3 Evaluation of tissue formation within the pores—Micro-CT analysis

**FIGURE 6:**
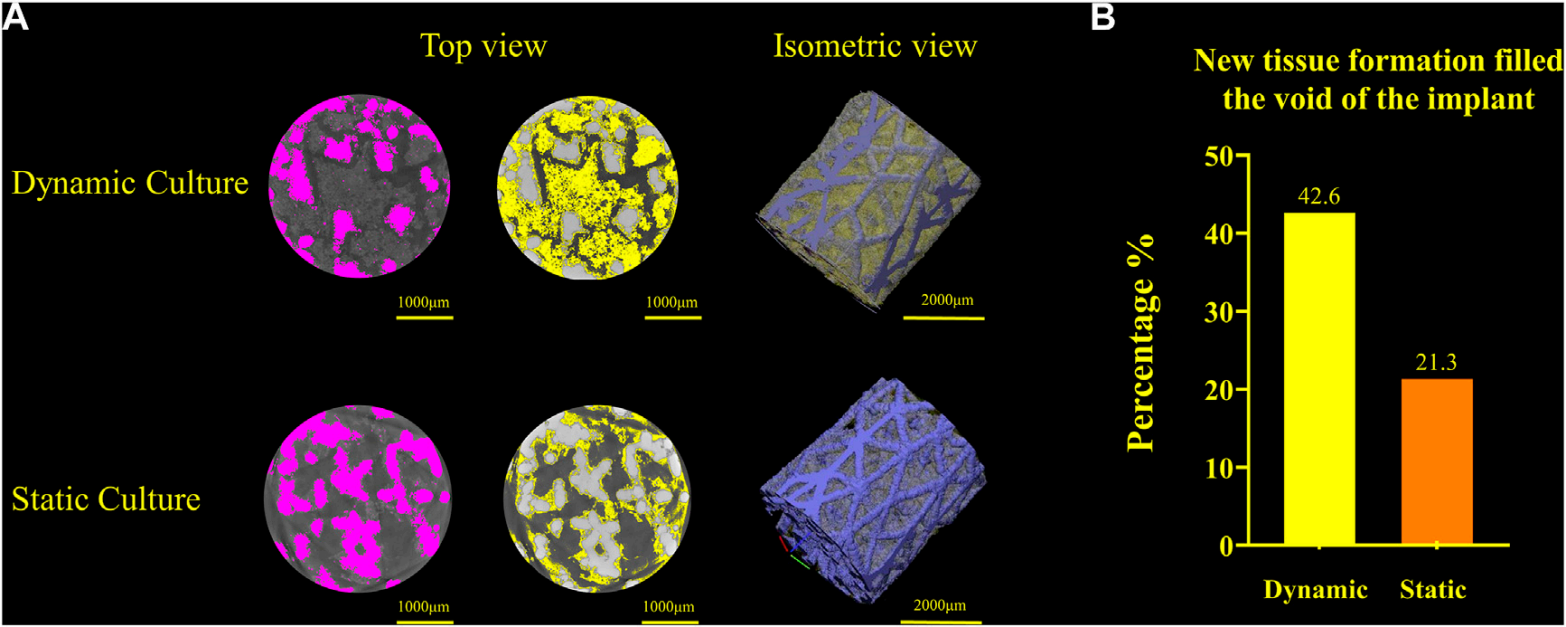
**(A)** Titanium implant micro-CT scans and 3D volume renderings for the dynamic culture (top) and the static culture (bottom) showing the segmented implant (pink, left), tissue (yellow, middle) and 3D render (titanium blue, tissue yellow, right). **(B)** Twice as much tissue volume, as a percentage of the void space, was detected for the dynamic culture condition.

Newly formed tissue was identified inside the porous structure with micro-CT for both culture conditions (Figure 6). The new tissue formation for the dynamic culture condition was twice that of the static culture, filling half the void space within the porous titanium implant.

### 3.4 Site specific bone remodelling—histology

**FIGURE 7:**
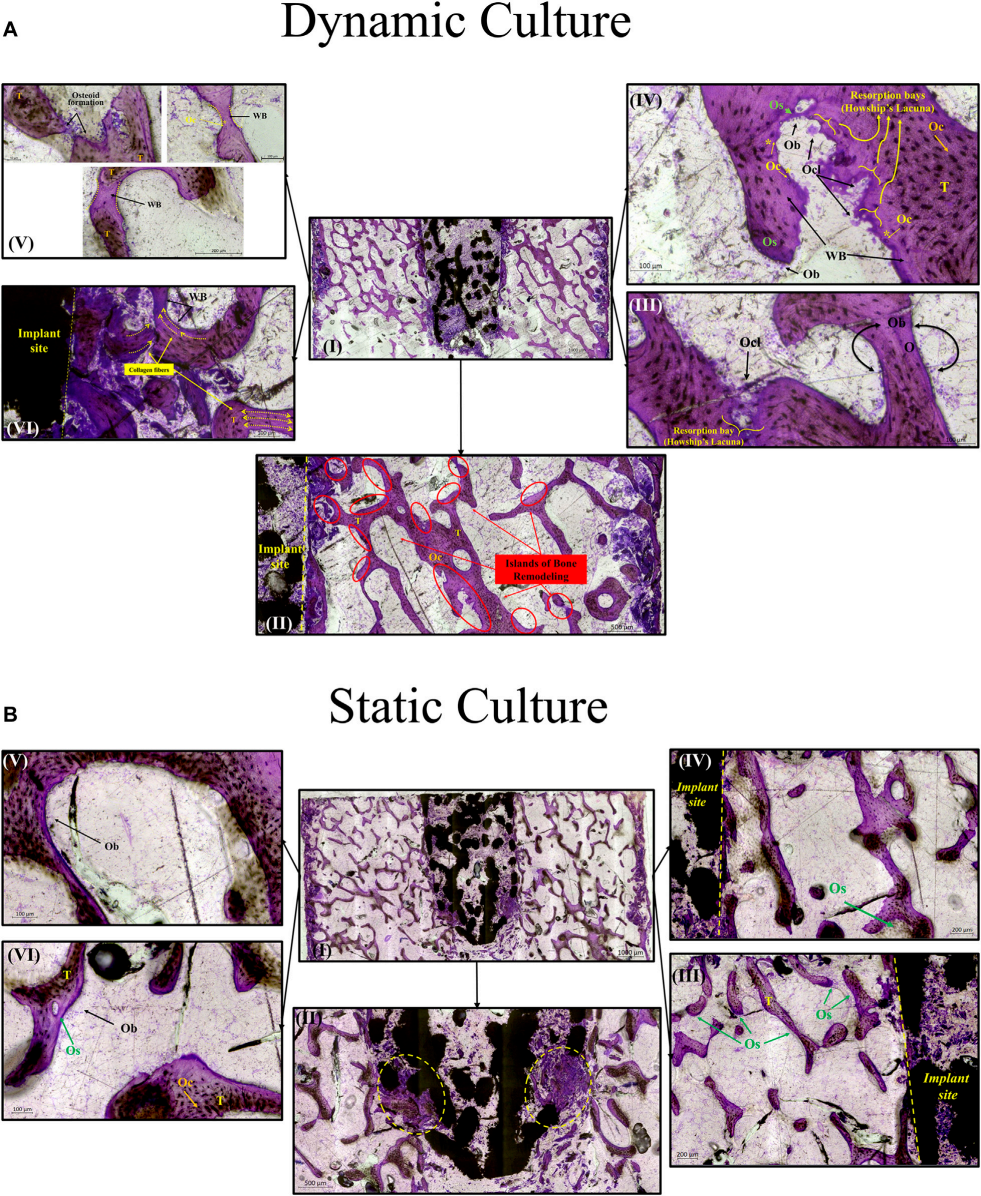
Transverse tissue sections stained with Paragon—Toluidine blue **(A)** *Ex-vivo* cancellous bone cultured for 21 Days within bioreactor (I) Cross section of the bone cylinder, (II) representative areas next to the implant fixation showing the newly formed bone tissue islands (red circles) on the existing trabeculae struts (T), (III) bone remodelling produced from osteoblasts (Ob) lining on both sides of the trabeculae, and bone resorption by osteoclasts (Ocl) occupying small depressions on the bone surface (Howship’s lacunae), (IV) areas of new osteoid formation (Os), woven bone (WB), bone resorption activity (Howship’s lacunae) and some newly formed Osteocytes (Oc*) together with mature osteocytes (Oc), (V) existing trabeculae struts with formation of new osteoid (Os) and woven bone formation bridging trabeculae struts with newly formed Osteocytes (Oc*) trapped inside, (VI) woven bone formation near the implant site with collagen fibers situated parallel in sheets and oriented opposite to the mechanical force acting on the sample. **(B)** Static culture for 21 Days (I) Cross section of the bone cylinders, (II) conical edge of the peg with circles highlighting mixed bone tissue (old mineralized bone and new connective tissue reformation), (III) left side and (IV) right side of bone next to the implant showing minor islands of osteoid (Os) formation, (V) and (VI) areas away from the implant with osteoblasts (Ob) forming new osteoid (Os). Scalebars are shown within the pictures.

For the dynamic samples, numerous osteoid formation seams and woven bone islands were observed (Figure 7A. I, II). Dense connective tissue formation was observed next to the implant, full of collagen (blue/pale blue). Formation of connective tissue, distributed within the pore network of the peg, was also noted. Osteoblasts with recognizable large, round nuclei and abundant basophilic cytoplasm that had deposited new osteoid (dark pink) on peri-implant bone surfaces, as well as deeper into the bone tissue (Figure 7A. III, IV, VI). Additionally, osteoclasts usually with four to seven nuclei, were observed, actively forming resorption bays and Howship’s lacunae (Figure 7A. III, IV). Active osteoblasts depositing new osteoid on trabeculae were also found, while other areas of mineralized bone struts had been bridged with new woven bone matrix (Figure 7A. V). Some of these osteoblasts were detected entrapped inside their own secreted bone matrix, differentiating to osteocytes (yellow asterisk, Figure 7A. IV, V). Collagen fibres situated parallel to each other like pillars, directing upwards and opposite to the compression force applied on the dynamically cultured samples, were observed in remodelling bone tissue close to the implant (Figure 7A. VI).

For static samples, osteoid seams were also observed (Figure 7B. I, III and IV). For these samples, connective tissue was formed predominantly at the tip of the implant where it was mixed with old bone tissue, (Figure 7B. II). Close to implant’s outer region, less area with new osteoid matrix formation was visible, and even less in the whole bone trabeculae network (Figure 7B. III, IV, V and VI).

### 3.5 Mechanical results

The characteristic loading times for the dynamic cultures was mostly of a short duration, with $T_{load}$ typically less than a second (Table 1).

**TABLE 1:** Mean ± S.D. characteristic loading times resulting from the cyclic displacement applied during the dynamic cultures; where I*:* $T_{load}<0.1\;s$ *, II:* $0.1\;s\leq T_{load}<1\;s$ *, III:* $1\;s\leq T_{load}<10\;s$ *, IV:* $10\;s\leq T_{load}$ .

I (%)	II (%)	III (%)	IV (%)
17 ± 11	60 ± 13	20 ± 16	3 ± 5

**FIGURE 8:**
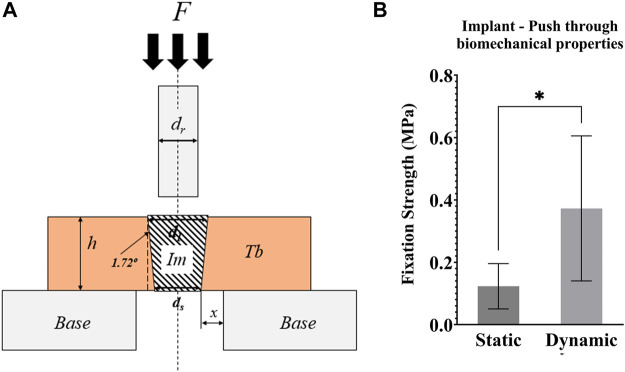
**(A)** Schematic representation of the push-through mechanical testing. Compression testing of all samples was performed after 21 days of culture. F = force applied to the implant with a compression platen; Im = implant; Tb = trabecular bone; h = height of the Tb; Base = support of the bone cylinder; x = clearance of hole in the support base; ds = small implant diameter; dl = large implant diameter; dr = push-through rod diameter < dl **(B)** Graph of the push-through fixation strength (MPa) comparing static and dynamic samples. *p <0.05, **p <0.01.

Push through mechanical testing was not possible for one sample, meaning that N = 6 dynamic and N = 5 static samples were tested. Dynamic samples were found to have 3x greater push-through fixation strength (*p* = 0.046, Figure 8B).

## 4 Discussion

The most important finding of this study is that the *ex vivo* model can be used to examine the early stages of tissue ingrowth (Figures 4–7), bone ongrowth (Figure 5) and bone remodelling (Figures 3, 4, 7) around porous implants. The use of a bioreactor with more physiological conditions (mechanical loading and fluid flow) led to greater bone remodelling around the implant and mineralised tissue formation within the implant. The bioreactor is inherently controllable and so could enable more repeatable preclinical tests, with scope to evaluate new technology under the exact same conditions allowing for more direct comparisons between novel, state-of-the-art and established implant fixation technologies.

The fluorescent intensity analysis found differences for the whole bone cross-section and in the bone adjacent to the implant (that was most mechanically stressed), however, the intensity analysis of the outer circumference of the bone (most exposed to fluid flow) did not reach statistical significance. This difference at the bone implant interface between static and dynamic samples was apparent along the length of the implant. This suggests that the daily cyclic mechanical loading applied to the implant had an important effect on the day 7 remodelling in our *ex vivo* model of implant fixation. Interestingly, the initial press-fit implantation in our study also affected early bone remodelling: the cross-sectional fluorescent imaging revealed day 7 calcium deposits directly below the implant in both the static and dynamic cultures. This finding that the mechanical loading has an important effect agrees with Birmingham et al. who analysed the effects of mechanical loading and fluid shear on trabecular remodelling and concluded that bone strain was likely the overall driving mechanical signal for new bone formation (Birmingham et al., 2016). Osteocytes have been measured to be the most mechanosensitive bone cells (Klein-Nulend et al., 1995). Two mechanisms have been proposed through which osteocytes could sense the strain that arises during physiological loading: fluid flow through the canaliculi, or pressurisation of the lacunar pores (Gardinier et al., 2010; Scheiner et al., 2016; Wittkowske et al., 2016; Estermann and Scheiner, 2018). For longer characteristic loading times (>8 s), fluid flow through the canaliculi could occur (Gardinier et al., 2010); however in this *ex vivo* study, only a small portion of the recorded loading times were of a such a duration (Table 1). Rather, the characteristic loading times in this experiment were much shorter, typically <1 s, indicating that lacunar pore pressurisation was likely to be the driver of osteocyte activity (Scheiner et al., 2016). The distribution of the characteristic loading times (Table 1) were physiological, similar to that experienced at the knee while ascending stairs and at the hip during gait (Scheiner et al., 2016).

The SEM analysis revealed many tissue deposits onto the surface of dynamically cultured samples (Figure 5). The tissue formed on the porous implants was visually similar to tissue-engineered woven bone grown on titanium *in vitro* from cultured osteoblasts (Chen et al., 2010), and the EDX analysis confirmed the presence of calcium and phosphorus. Previous *ex vivo* research utilising SEM analyses also found that application of cyclic loading plus fluid flow in a bioreactor led to increased extracellular matrix transfer to an implant surface indicative of the early stages of implant bone ongrowth (Dua et al., 2021). In their study, the amount of matrix was highest after 7 weeks of culture. Our static culture led to visually similar level of matrix after 3 weeks, with even more observed in the dynamic model. This may be a result of the amount of mechanical stress induced in the bone because of the press-fit fixation, but it could also be a result of the different pore sizes and surface topography of the stochastic porous implant used in our study leading to an increased rate of bone ongrowth.

Woven bone formation was observed in the trabecular network and mineralisation was detected inside the dynamically cultured implants (Figure 7). Bone remodelling and tissue maturation *in vivo* occurs over a period of months (Kohli et al., 2018), rather than days, and hence a longer-term culture would be required to assess the extent to which tissue ingrowth can remodel to a fully osseointegrated implant. After 6 weeks *in vivo* in an ovine femoral condyle model, a similarly designed, but larger diameter, stochastic porous implant had 11% bone ingrowth, with the outer porous struts encased in woven bone (Ghouse et al., 2019). A rat model of commercially pure titanium implants found that after 1 week, the implant-bone interface was characterized by fibrous tissue, at 2 weeks, there was little evidence of bone-implant contact, with the appearance of fragments with fibrous layers adjacent to the implant, like that observed in our model, and by 4 weeks, bone-implant contact had nearly trebled, with newly formed woven bone attached to the implant (Zhang et al., 2010). The remodelling pathway observed in our model appears like the early stages observed *in vivo* but at a slower rate. A similar observation was also made by others (Dua et al., 2021) The dynamic culture led to more remodelling, refinement of the fluid flow and loading conditions may thus allow for the rate of remodelling to be accelerated further.

*Ex vivo* models simulate blood supply with fluid flow but do not currently model angiogenesis which is required for remodelling *in vivo* (Kohli et al., 2018). Thus, the model may be best applied to narrow the design space and screen how new technology influences the bone remodelling pathway before final proof-of-concept in an *in vivo* model. Adoption of *ex vivo* models for early research (technology readiness levels 1–4) would still have a tangible ethical impact and financial impact (the cost of the protocol is one to two orders of magnitude lower than that of a large animal model). The level of control enabled will also facilitate comparison across research centres and years. Cell viability assessment for the *ex vivo* bone was found to be more challenging than for cell culturing experiments: the common microscopy approach (Calcein AM and Ethidium Homodimer with confocal microscopy) was less effective as the dye did not penetrate most of the cells, preventing accurate quantitative analysis. This was likely a consequence of the abundant bone extracellular matrix that is inherent to *ex vivo* bone covering the cells. Others have previously reported live/dead images, but did not include quantitative analyses (Dua et al., 2021). Qualitatively, they described fewer dead cells after 7 weeks of dynamic culture. Early *ex vivo* bone research used *in situ* histochemical analysis to demonstrate that 60% of osteocytes are viable after 3 weeks in loaded samples (David et al., 2008). Cell metabolic activity measurements could also provide insight into viability, with prior research suggesting no change in viability for *ex vivo* bone from days 4–5 to days 21–22 (Birmingham et al., 2015). Media measurements could also be used to quantify the levels of formation/resorption markers at regular time intervals throughout the experiment, while development of a protocol to enable RT-PCR measurements could provide additional quantitative insight from an end-test analysis. Bone samples were extracted from both condyles, and bone density and properties may have differed between samples at day zero (Munford et al., 2020). We randomized the location for which static and dynamic samples were obtained to minimise these effects when comparing culture conditions. Further, our imaging analyses of bone remodelling were not dependent on bone density/volume. For our mechanical testing, we chose to test fixation as a push-through test rather than a push-out test to capture the effects of bone remodelling around the tapered implant, in addition to any mechanical bone-implant fixation. A pull-out test could be used in future research to focus purely on the bone-implant fixation strength. Modelling the initial press-fit fixation and the subsequent daily cyclic loading would also be valuable future work to predict the bone stress-strain state for this bioreactor model of implant fixation. Linking these predictions to previous poromicromechanics simulations (Scheiner et al., 2016) would lead to further insight into the stimulus experienced by the osteocytes. Finally, it is possible that the loading could have affected the distribution of the fluorochrome strains even in the absence of active bone formation. This effect is likely to be small as there was good stain penetration throughout the samples in both the dynamic and static samples, and the histology analysis provided complementary evidence of osteoid formation, and the histology and backscatter SEM analyses provided complementary evidence of mineralisation within the implant pores.

In conclusion, *ex vivo* bone models enable the analysis of tissue remodelling onto, into and around implants in the lab. While static culture conditions exhibited some characteristics of bony adaptation to implantation, controlling the fluid flow and mechanical loading environment with a bioreactor to simulate physiological conditions led to an accelerated response.

## Data Availability

The original contributions presented in the study are included in the article/Supplementary Material, further inquiries can be directed to the corresponding author.
